# Supplementary material for: Structural and Functional Annotation of Transposable Elements Revealed a Potential Regulation of Genes Involved in Rubber Biosynthesis by TE-Derived siRNA Interference in Hevea brasiliensis
Source: Int J Mol Sci. 2020 Jun 13;21(12):4220. doi: 10.3390/ijms21124220 (PMC7353026; doi:10.3390/ijms21124220)
Supplement: Supplementary file 1 [file ijms-21-04220-s001.zip › supplementary Figure S3.pptx]

## Slide 1
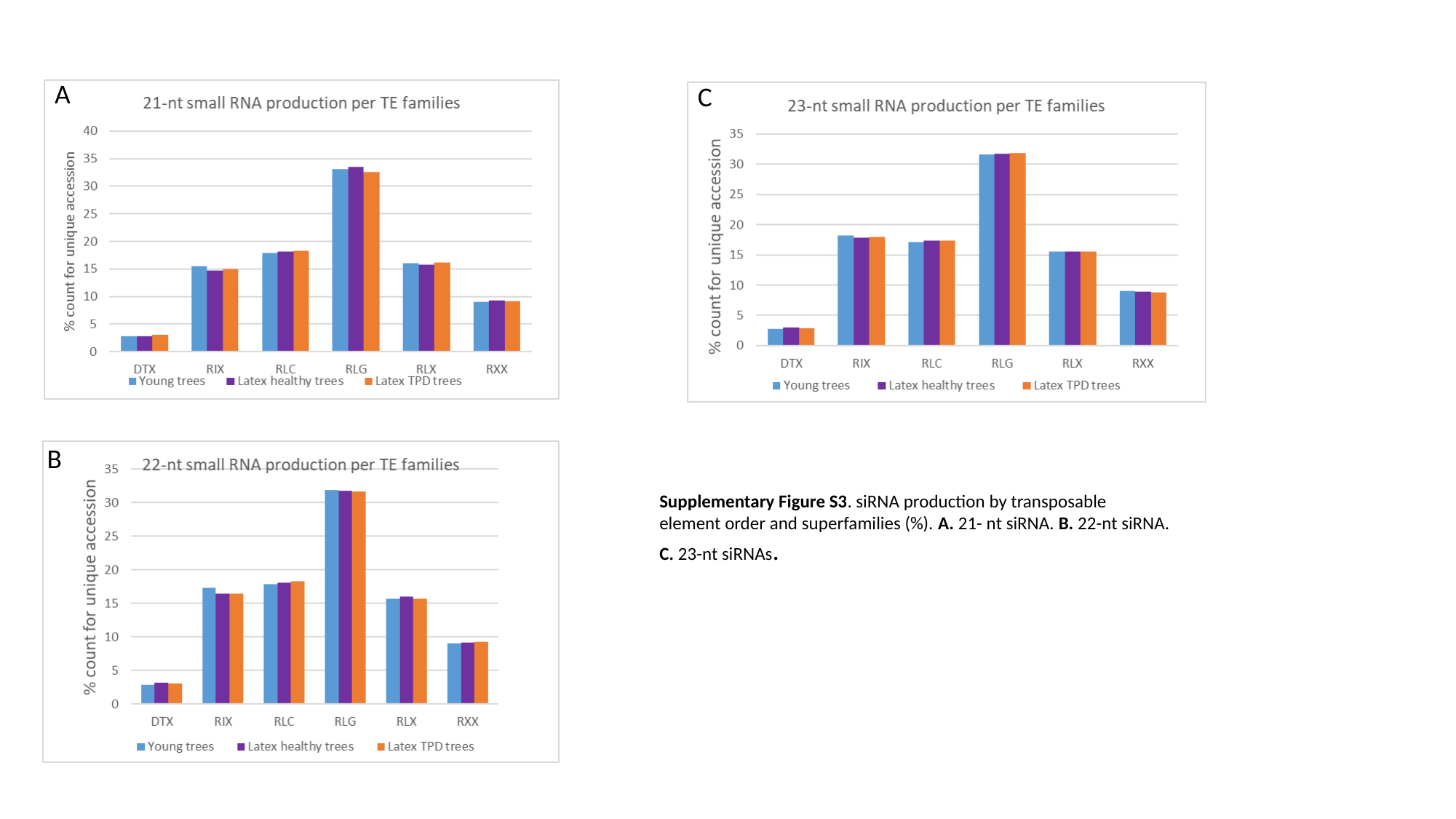

A
C
B
Supplementary Figure S3. siRNA production by transposable element order and superfamilies (%). A. 21- nt siRNA. B. 22-nt siRNA. C. 23-nt siRNAs.
