# Supplementary figures and images for: Structural and Functional Annotation of Transposable Elements Revealed a Potential Regulation of Genes Involved in Rubber Biosynthesis by TE-Derived siRNA Interference in Hevea brasiliensis
Source: Int J Mol Sci. 2020 Jun 13;21(12):4220. doi: 10.3390/ijms21124220 (PMC7353026; doi:10.3390/ijms21124220)

*A*

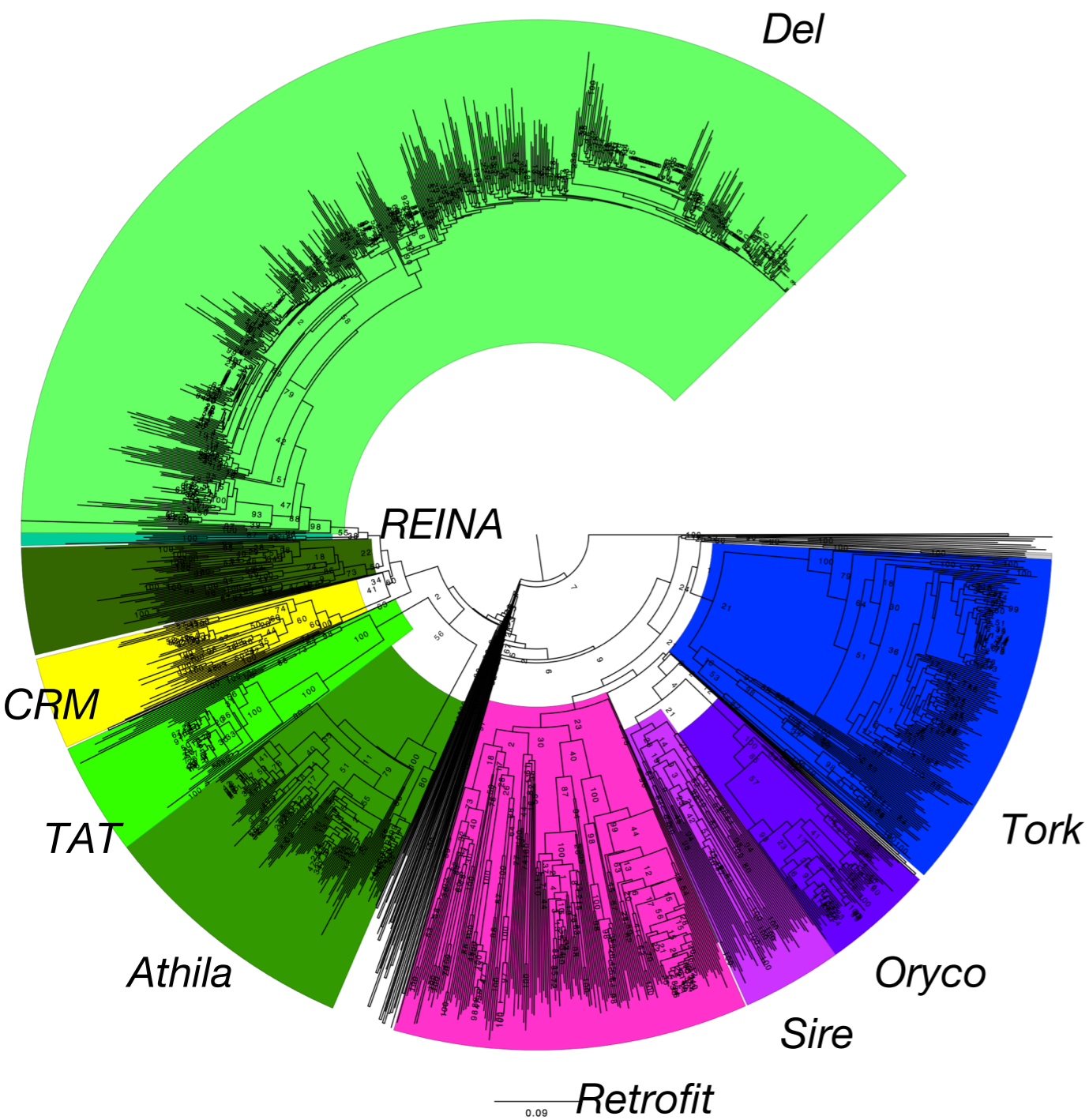

*B*

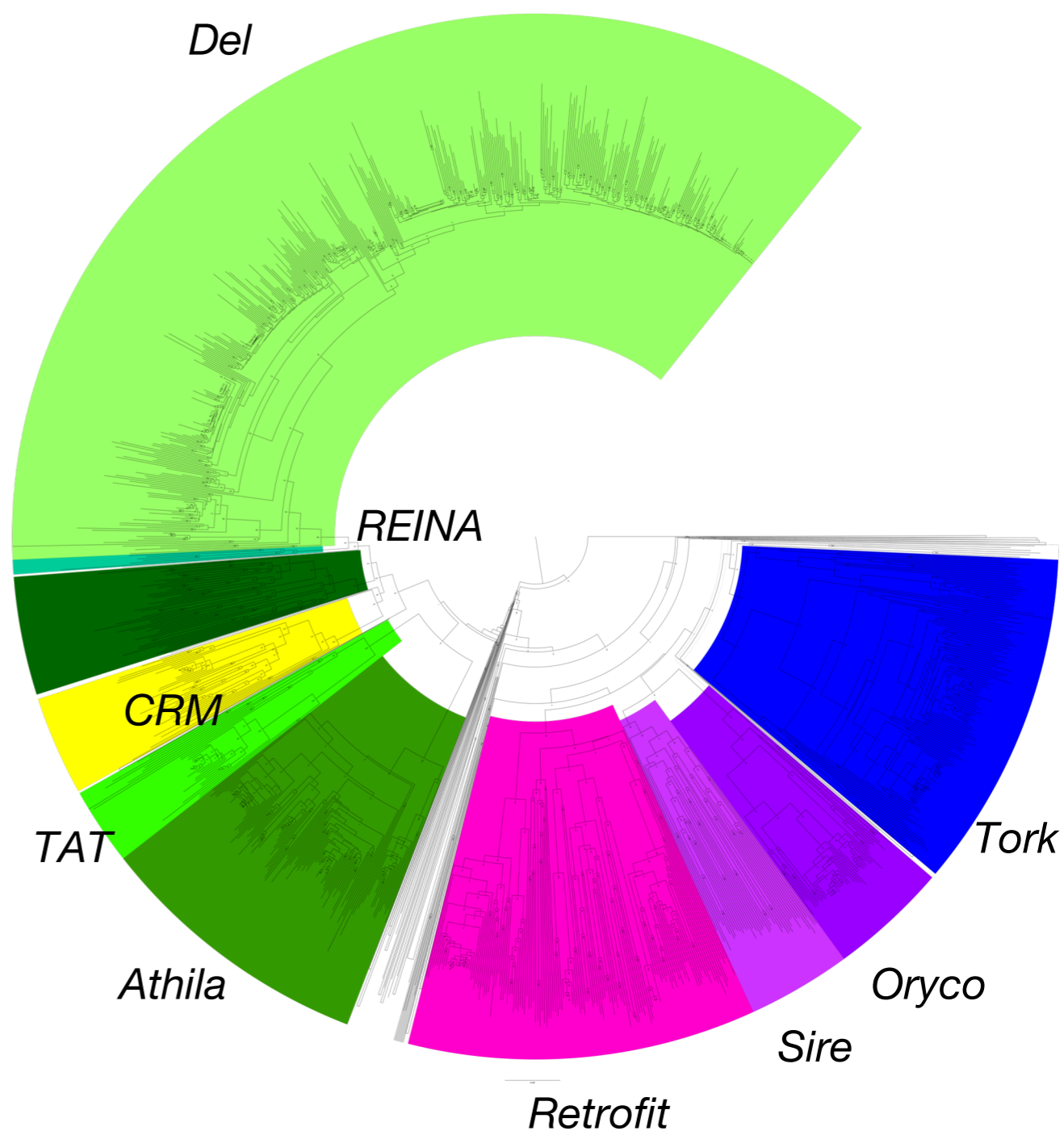

Supplement: Supplementary file 1 [file ijms-21-04220-s001.zip › Supplementary Figure S2.pdf]
