# Supplementary material for: Structural and Functional Annotation of Transposable Elements Revealed a Potential Regulation of Genes Involved in Rubber Biosynthesis by TE-Derived siRNA Interference in Hevea brasiliensis
Source: Int J Mol Sci. 2020 Jun 13;21(12):4220. doi: 10.3390/ijms21124220 (PMC7353026; doi:10.3390/ijms21124220)
Supplement: Supplementary file 1 [file Supplementary Figure S1 avril 2020.pdf › Supplementary Figure S1 avril 2020.pdf]

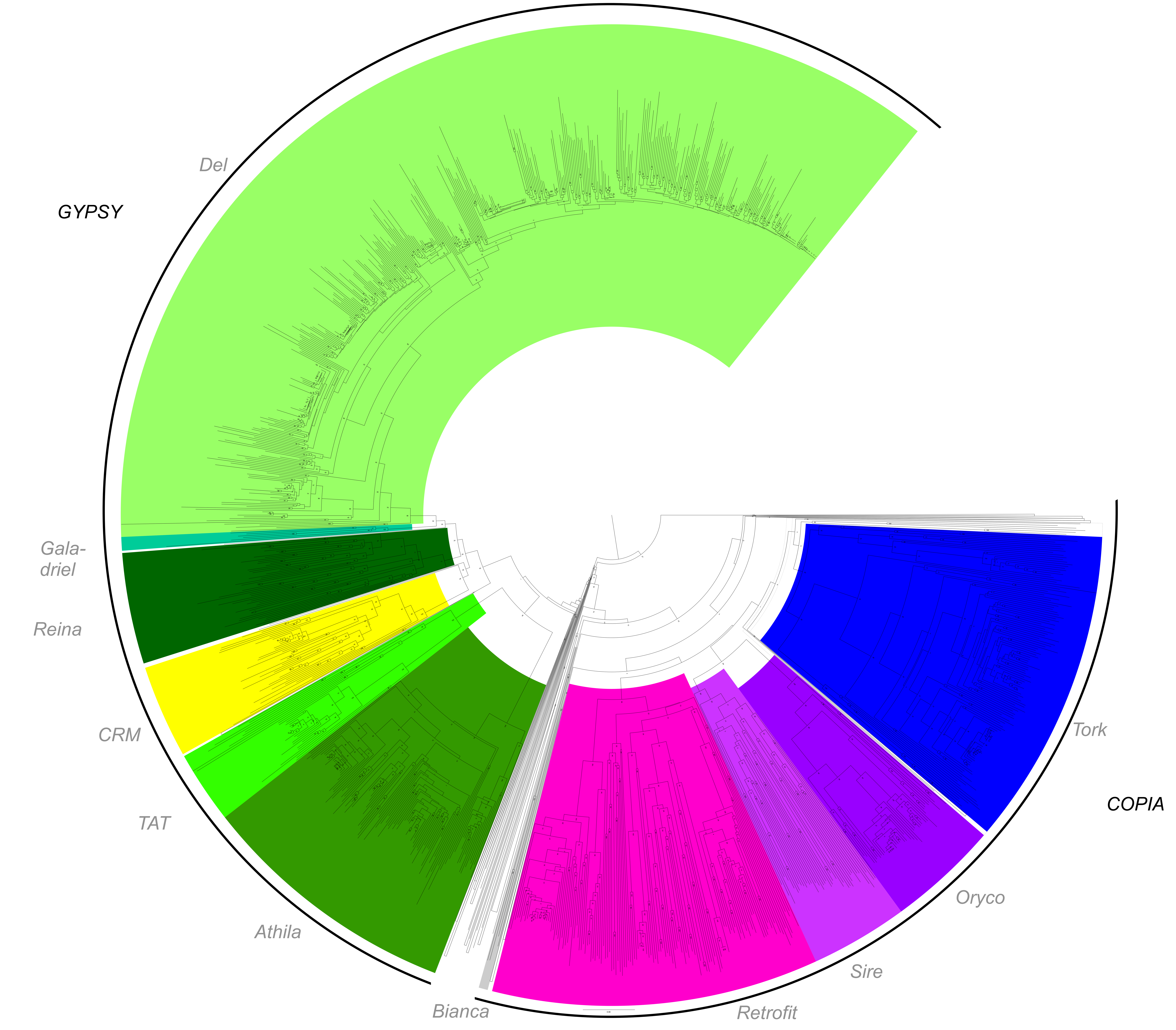
GYPSY
Del
Gala-
driel
Reina
CRM
TAT
Athila
Bianca
Retrofit
Sire
Oryco
Tork
COPIA
